# Traditional Chinese medicine as a potential barrier-oriented sensitization strategy for immune checkpoint blockade in microsatellite-stable colorectal cancer: from resistance mechanisms to translational validation

**DOI:** 10.3389/fimmu.2026.1874066

**Published:** 2026-06-29

**Authors:** Dawei Wang, Yawei Wang, Xuan Wang

**Affiliations:** 1 Department of Medical Oncology, The Third Affiliated Hospital of Shandong First Medical University (Affiliated Hospital of Shandong Academy of Medical Sciences), Jinan, Shandong, China; 2 Innovative Institute of Chinese Medicine and Pharmacy, Shandong University of Traditional Chinese Medicine, Jinan, Shandong, China

**Keywords:** gut microbiota, immune checkpoint inhibitors, immunometabolism, microsatellite stable colorectal cancer, traditional Chinese medicine, tumor microenvironment

## Abstract

**Background:**

Microsatellite-stable colorectal cancer (MSS-CRC) is refractory to immune checkpoint inhibitors (ICIs), mainly because of low tumor immunogenicity, impaired antigen presentation, immune exclusion, and myeloid- or Treg-dominated immunosuppression. Traditional Chinese medicine (TCM), characterized by multi-component and multi-target regulation, may provide complementary approaches for modulating these resistance barriers. This review summarizes the rationale, evidence, and translational requirements for integrating TCM with ICI-based therapy in MSS-CRC.

**Purpose:**

To evaluate the potential of TCM to regulate immune-resistance mechanisms in MSS-CRC and to propose a barrier-oriented framework for rational TCM–ICI combination strategies.

**Methods:**

A structured narrative review was conducted in PubMed, Web of Science, Embase, Scopus, CNKI, and Wanfang for studies published from January 2019 to March 2026. Searches covered MSS/pMMR CRC, immune checkpoint blockade, TCM interventions, tumor microenvironment, microbiota, immunometabolism, syndrome patterns, active constituents, and herbal quality control. Eligible studies addressed MSS/pMMR CRC immunotherapy, TCM or phytomedicine-based interventions, or immune-resistance mechanisms. Direct MSS/pMMR CRC studies involving ICI combinations were prioritized, while broader CRC, cross-cancer, pharmacological, and computational studies were used as supportive evidence.

**Results:**

Selected TCM interventions may improve ICI responsiveness in MSS-CRC when matched to specific resistance barriers. Representative interventions include Zhenqi Fuzheng Granules, Shenqi Yichang Formula, Gegen Qinlian Decoction, modified Shenling Baizhu San, Changweiqing, and electroacupuncture-related evidence. These interventions have been linked to immunometabolic remodeling, dendritic-cell mitophagy and antigen presentation, microbiota-driven TME remodeling, Tfh–B-cell interactions, and STING-dependent immune activation. However, direct formula-specific clinical evidence for ICI sensitization in molecularly defined MSS-CRC remains limited. Clinical-translational evidence includes an early phase II study of electroacupuncture combined with fruquintinib and sintilimab in refractory MSS-mCRC, whereas most herbal-formula evidence remains preclinical or indirect. We therefore propose matching TCM interventions and active ingredients to dominant immune-resistance phenotypes and integrating syndrome-informed host-state stratification. These hypotheses require validation using causal assays, organoid-immune co-culture, single-cell/spatial omics, pharmacological standardization, and biomarker-enriched clinical trials.

**Conclusion:**

TCM may serve as a complementary, mechanism-informed candidate sensitization strategy for MSS-CRC immunotherapy, but it is not an established standard combination approach. Future translation requires formula-specific and component-level validation, reproducible immune and microbiota-metabolic biomarkers, TCM syndrome assessment, herbal quality control, safety monitoring, and clinically meaningful outcomes.

## Graphical Abstract

**Figure:**
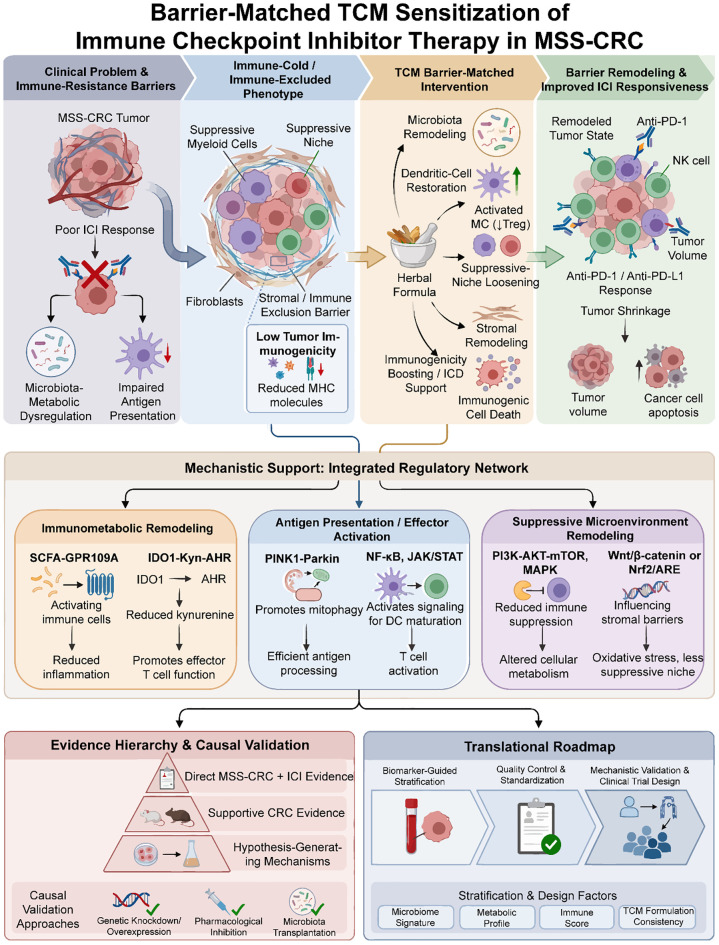

## Highlight

- TCM as a Potential Sensitization Strategy: This review examines how Traditional Chinese Medicine (TCM) may enhance MSS-CRC responsiveness to immune checkpoint inhibitors (ICIs) by targeting multi-layered resistance barriers and by incorporating TCM syndrome-informed host-state stratification.
- Resistance Barriers in MSS-CRC: Resistance arises from tumor immunogenicity, impaired antigen presentation, immune exclusion, myeloid/Treg-dominant suppression, and microbiota-metabolism dysregulation, which limit ICI efficacy.
- Barrier-Oriented and Syndrome-Informed TCM Interventions: The review highlights mechanism-matched formulas, active constituents and integrative modalities, including Zhenqi Fuzheng Granules, Shenqi Yichang Formula, Gegen Qinlian Decoction, Changweiqing and electroacupuncture-related evidence, while linking these interventions to immune barriers and TCM syndrome patterns.
- Evidence Gaps and Future Directions: Current evidence remains uneven. Direct herbal-formula plus ICI clinical evidence in molecularly confirmed MSS-CRC is scarce, and available findings require causal validation, component-level characterization, standardized quality control and biomarker-enriched trials.
- Clinical Translation Framework: The article proposes a barrier-matched and syndrome-informed framework for future trials, integrating immune, microbiota-metabolic, spatial, clinical and TCM syndrome variables to inform future ICI-based strategies in MSS-CRC.

## 1 Introduction

Immune checkpoint inhibitors (ICIs) have reshaped the therapeutic landscape of colorectal cancer (CRC). Their clearest benefit has been observed in microsatellite instability-high (MSI-H) and deficient mismatch repair (dMMR) tumors, in which durable responses can occur. The randomized KEYNOTE-177 trial established pembrolizumab as a benchmark first-line immunotherapy option for MSI-H/dMMR metastatic CRC and reinforced mismatch-repair status as a key predictor of ICI benefit (1). However, most CRC cases are microsatellite stable (MSS) and proficient mismatch repair (pMMR). These tumors generally derive little benefit from anti-PD-1/PD-L1 monotherapy (2–7). Overcoming primary immune resistance in MSS-CRC therefore remains a major challenge in CRC immunotherapy.

The limited responsiveness of MSS-CRC to ICIs cannot be explained by checkpoint expression alone. It reflects a multilayered resistance architecture involving low tumor immunogenicity, impaired antigen presentation, immune exclusion, suppressive myeloid/Treg-dominant niches, and microbiota- and metabolism-associated abnormalities (4, 8–12). Current sensitization strategies have combined ICIs with chemotherapy, anti-angiogenic or multikinase agents, radiotherapy, MEK inhibition, or other targeted therapies. However, clinical results remain heterogeneous. IMblaze370 did not improve survival with atezolizumab plus cobimetinib or atezolizumab alone compared with regorafenib (13). Other regorafenib- or multikinase-based ICI combinations have shown subgroup-dependent signals, often influenced by metastatic pattern, especially liver involvement (13–16). These findings support biologically stratified strategies rather than empirical all-comer combinations.

In this context, traditional Chinese medicine (TCM) has attracted interest as a potential complementary strategy for modulating immune-resistance barriers in MSS-CRC (17). Its multi-component, multi-target, and system-level features may allow coordinated regulation of tumor cells, immune compartments, metabolic pathways, and the gut microecological environment. A clinically relevant TCM framework should also consider syndrome differentiation. TCM syndromes may reflect host immune fitness, inflammatory burden, gut dysfunction, metabolic imbalance, and treatment tolerance. At present, relatively direct evidence is concentrated in a limited number of interventions, including Shenqi Yichang Formula, Changweiqing, modified Shenling Baizhu San, Zhenqi Fuzheng Granules, and Gegen Qinlian Decoction (18–22). Beyond these examples, many claims still rely on supportive CRC evidence, active-ingredient studies, or microbiota/immunometabolism-related mechanisms. The therapeutic boundaries, responsive populations, and safety profile of TCM combined with ICIs in MSS-CRC therefore require rigorous validation.

This review adopts a barrier-oriented and syndrome-informed framework. It links MSS-CRC resistance mechanisms with mechanism-matched TCM interventions, representative active constituents, evidence grading, and translational validation. Compared with reviews centered on individual formulas, isolated pathways, or the broad concept of “enhancing efficacy and reducing toxicity,” this review uses resistance barriers as the organizing principle. It further emphasizes the matching of formulas or components to specific biological contexts and separates direct sensitization evidence from supportive or hypothesis-generating mechanisms.

To support interpretive transparency, this review used a structured narrative approach rather than a systematic review or meta-analysis. PubMed, Web of Science Core Collection, Embase, Scopus, CNKI, and Wanfang were searched for studies published from January 2019 to March 2026. Search terms covered MSS/pMMR CRC, immune checkpoint blockade, TCM-related interventions, tumor microenvironment, microbiota, immunometabolism, syndrome patterns, active constituents, and herbal quality control. Direct MSS/pMMR CRC studies involving ICI-based combinations were prioritized. Broader CRC, cross-cancer, pharmacological, and computational studies were considered supportive when they were relevant to immune-resistance mechanisms, active constituents, syndrome stratification, or formula quality control.

## 2 Biological barriers underlying resistance of MSS-CRC to immune checkpoint blockade

The poor responsiveness of MSS-CRC to immune checkpoint blockade reflects a layered resistance architecture. This architecture involves tumor cell-intrinsic immune evasion, an immunosuppressive tumor microenvironment (TME), and microbiota-metabolism dysregulation. It cannot be explained by isolated abnormal pathways alone. Tumor cells determine whether antitumor immunity can be initiated. The TME determines whether effector immunity can infiltrate and persist. The microbiota-metabolic milieu acts as a peripheral amplifier that stabilizes immune resistance.

**Figure 1:**
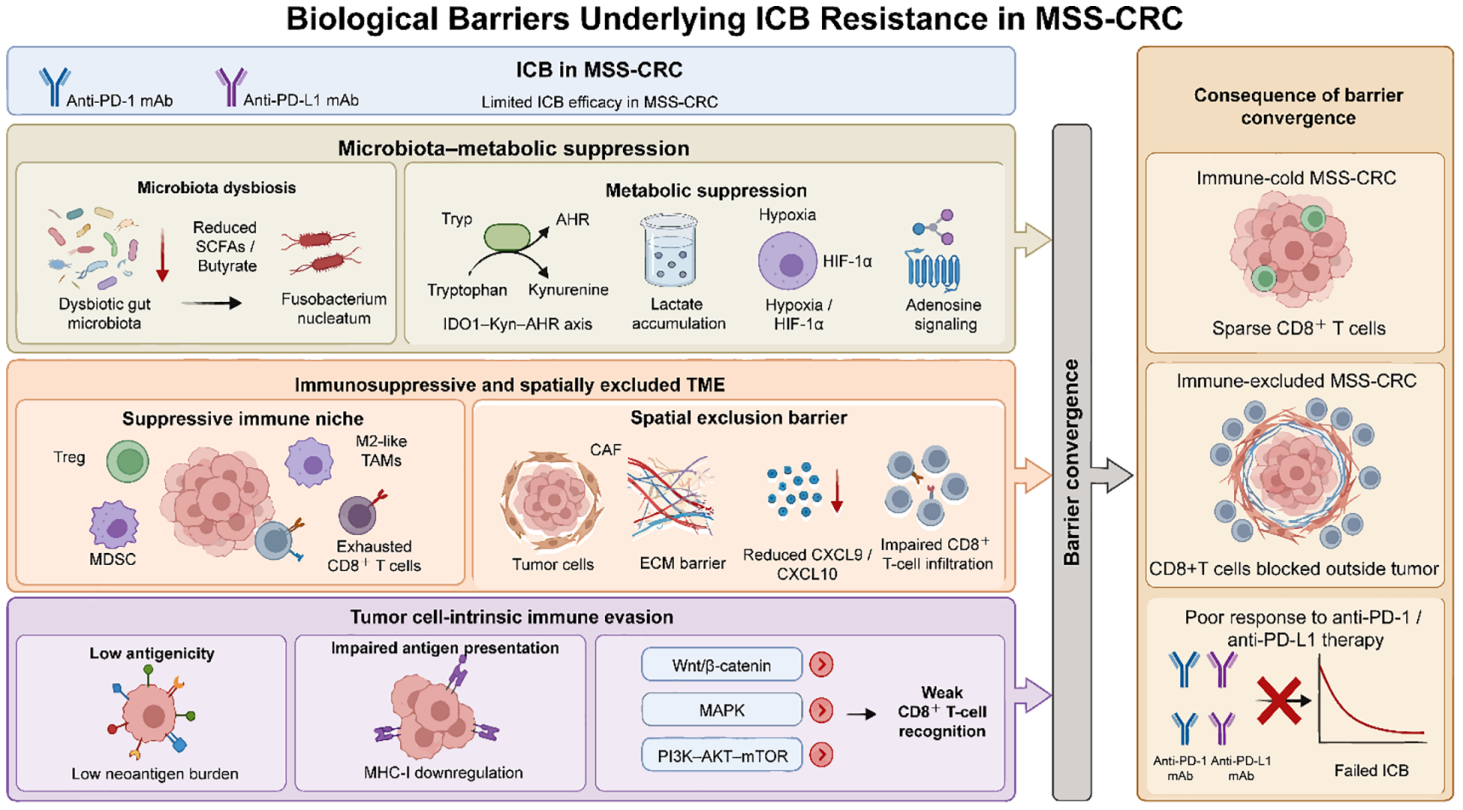
Biological barriers underlying ICB resistance in MSS-CRC. MSS-CRC shows limited responsiveness to anti-PD-1/anti-PD-L1 therapy due to convergent biological barriers, including microbiota–metabolic suppression, an immunosuppressive and spatially excluded tumor microenvironment, and tumor cell-intrinsic immune evasion. These barriers collectively reduce antigenicity and antigen presentation, weaken CD8^+^ T-cell recognition and infiltration, reinforce suppressive immune niches, and promote immune-cold or immune-excluded tumor states, ultimately contributing to poor ICB efficacy. CAF, cancer-associated fibroblast; ICB, immune checkpoint blockade; MSS-CRC, microsatellite-stable colorectal cancer; TME, tumor microenvironment.

For translational purposes, these biological layers can be converted into five operational barriers. These include microbiota-metabolite dysregulation, antigen-presentation failure, myeloid/Treg-dominant suppressive niches, immune exclusion/stromal restriction, and low tumor immunogenicity. These barriers are used in this review to map TCM interventions and define barrier-matched validation strategies (**Figure 1**).

### 2.1 Tumor cell-intrinsic immune evasion: low immunogenicity and impaired antigen presentation

A major basis for intrinsic resistance of MSS-CRC to ICIs is insufficient tumor immunogenicity. Compared with MSI-H/dMMR tumors, MSS tumors usually have a lower neoantigen burden and weaker antigen presentation. As a result, they are less readily recognized by the immune system (11, 12). This phenotype is often characterized by downregulation of major histocompatibility complex class I (MHC-I), impaired antigen processing and presentation, and reduced exposure of immunogenic neoantigens (11). Together, these changes weaken CD8+ T-cell recognition and cytotoxic activity. They contribute to a state of immune ignorance.

Persistent oncogenic signaling further consolidates this immune-evasive phenotype. Aberrant Wnt/beta-catenin activation has been implicated in MSS-CRC progression and restricted T-cell recruitment (11). Dysregulated MAPK and PI3K-AKT-mTOR signaling may also promote immunosuppressive mediators, such as vascular endothelial growth factor and interleukin-10. These pathways can further support a metabolically suppressive microenvironment (11, 12).

Thus, low immunogenicity in MSS-CRC should not be viewed as a static deficiency. It is a dynamic phenotype coupled to oncogenic signaling, metabolic rewiring and immunosuppressive mediator production. In other words, resistance is not merely caused by too few antigens. It is also maintained by an active context that is unfavorable for immune recognition and activation (12).

Accordingly, tumor cell-intrinsic immune evasion functions as an upstream node in the MSS-CRC resistance network. It raises the threshold for immune activation required for ICIs to exert therapeutic benefit. It also cooperates with downstream microenvironmental suppression to maintain a non-responsive phenotype. This helps explain why checkpoint blockade alone rarely reproduces in MSS tumors the benefit observed in MSI-H disease.

### 2.2 The immunosuppressive TME: suppressive immune cells, stromal barriers and spatial exclusion

Tumor cell-intrinsic immune evasion makes antitumor immunity difficult to initiate. The immunosuppressive TME then limits immune persistence and amplification. MSS-CRC is not simply devoid of immune cells. Instead, it often shows weak effector T-cell recruitment, restricted intratumoral entry and rapid functional exhaustion. Reduced expression of T-cell-attracting chemokines, such as CXCL9 and CXCL10, may limit cytotoxic T-lymphocyte infiltration into the tumor core (10). Even when T cells enter the lesion, they may express inhibitory receptors such as PD-1, TIM-3 and LAG-3. MSS-CRC is therefore better described as an immune-excluded and functionally inactivated state rather than as a static, cell-empty cold tumor (9).

Several immunosuppressive cell populations form the backbone of this resistant network. Regulatory T cells (Tregs), M2-like tumor-associated macrophages (TAMs) and myeloid-derived suppressor cells (MDSCs) are commonly enriched in MSS-CRC. These cells suppress effector T-cell proliferation and cytotoxicity. Key mediators include transforming growth factor-beta, interleukin-10 and arginase. These suppressive populations do not act independently. They reinforce one another through cytokine networks, metabolic competition and signaling crosstalk, thereby forming a stable immunosuppressive niche (10).

Stromal organization further strengthens immune resistance. In many MSS-CRC tumors, immune cells may be present but spatially restricted. They are often retained in stromal regions, invasive margins or CAF-rich compartments rather than entering tumor nests. This spatial restriction limits productive immune attack even when immune-cell abundance appears increased. Therefore, the MSS-CRC TME should be understood as a spatially organized suppressive system. It is maintained by inhibitory immune cells, stromal barriers and suppressive mediators, rather than by a single abnormal pathway.

Overall, the MSS-CRC TME creates a steady-state environment that limits T-cell infiltration, persistence and function. This multicomponent and self-reinforcing network helps explain why MSS-CRC remains a typical low-response subtype under immune checkpoint blockade.

### 2.3 Gut microbiota and immunometabolism: peripheral amplification of immune resistance

The gut microbiota and microbiota-shaped immunometabolism form a peripheral regulatory layer of MSS-CRC immune resistance. CRC develops in close contact with the intestinal microecological environment. Therefore, local CRC immunity is not determined only by cancer cells and immune cells. It is also shaped by microbial composition, microbial metabolites and mucosal barrier integrity (23).

Current evidence suggests that MSS/pMMR CRC is often accompanied by microbial dysbiosis and abnormal immunoregulatory metabolites. Depletion of short-chain fatty acids (SCFAs), especially butyrate, has received particular attention (24, 25). Reduced SCFA signaling may impair epithelial barrier homeostasis. It may also alter the functional states of dendritic cells, macrophages and T cells. These changes can further support an immunosuppressive TME (19, 24).

Several metabolic pathways amplify the effects of microbial dysbiosis. The IDO1-kynurenine-AHR axis is a representative example (26). In this pathway, indoleamine 2,3-dioxygenase 1 (IDO1) converts tryptophan into kynurenine. Kynurenine then activates the aryl hydrocarbon receptor (AHR), promotes Treg differentiation and suppresses CD8+ T-cell function (9). This pathway may interact with other metabolic suppressive signals. These include hypoxia, lactate accumulation, HIF-1alpha activation, CD73 activity and adenosine A2A receptor signaling. Together, these signals stabilize a metabolically suppressive phenotype.

Pathobiont-associated taxa may also participate in this suppressive network. For example, Fusobacterium nucleatum has been reported to promote MDSC and Treg enrichment through pathways such as Toll-like receptor 4 and NF-kappaB signaling (27). Accordingly, immune resistance in MSS-CRC is not restricted to the tumor bed. It reflects a coupled process involving local immune suppression, microbial dysbiosis and systemic metabolic disturbance (9). This cascade helps explain why single-agent checkpoint blockade rarely produces substantial benefit in MSS-CRC.

Taken together, gut microbiota-immunometabolic disruption acts as a peripheral amplifier of the MSS-CRC resistance network. It shapes local immune-cell composition and remodels the metabolic background in ways that are unfavorable for ICI activity. Mechanistic interpretations of MSS-CRC immune resistance should therefore integrate tumor-intrinsic, TME-related and microbiota-metabolic factors rather than focusing only on tumor cells or checkpoint molecules (**Figure 1**).

## 3 Barrier-based mechanisms of TCM-mediated resistance-barrier remodeling in MSS-CRC

Candidate TCM-ICI strategies in MSS-CRC should be evaluated through three linked questions. First, which resistance barrier is being targeted? Second, which biomarker defines that barrier? Third, what validation is required to establish causality? TCM-related interventions can be mapped onto five overlapping but analytically distinct barriers: microbiota-metabolite dysregulation, antigen-presentation failure, myeloid/Treg-dominant suppressive niches, immune exclusion/stromal restriction, and low tumor immunogenicity.

### 3.1 Microbiota-metabolite barrier: from dysbiosis to functional metabolic remodeling

Microbiota-metabolite dysregulation is a plausible entry point for TCM-based ICI sensitization. The intestine is both the anatomical site of CRC and a regulator of local and systemic immunity. In MSS-CRC, dysbiosis may sustain chronic inflammation, impair epithelial barrier function and reduce beneficial metabolites. It may also reinforce suppressive circuits involving IDO1-kynurenine-AHR signaling, lactate, adenosine, bile-acid remodeling and polyamine metabolism. The key translational question is whether a formula can shift microbial metabolic output toward an immune-permissive state.

Zhenqi Fuzheng Granules provide one clear example of microbiota-metabolic remodeling. They have been associated with enrichment of butyrate-producing bacteria and increased SCFAs in serum and tumor tissue. These changes may activate the SCFA-GPR109A axis, inhibit AKT/mTOR/HIF-1alpha signaling, reduce Warburg-like metabolism and improve PD-1 antibody efficacy in CRC models (21). These findings support a model in which a formula reconditions the metabolic background in which dendritic cells, macrophages and cytotoxic T cells operate. In this model, the tumor ecosystem becomes more permissive to checkpoint blockade.

Gegen Qinlian Decoction (GQD) provides another important example. In CT26 MSS-type CRC models, GQD enhanced the effect of PD-1 blockade by remodeling the gut microbiota and the TME (22). Related microbiome work showed that microbial changes can influence glycerophospholipid metabolism and regulate the therapeutic potential of PD-1 antibody in MSS-type CRC tumor-bearing mice (28). These findings are relevant to MSS-CRC because they connect microbial remodeling with immune-cell activation, including increased CD8+ T-cell-related antitumor responses. However, the clinical translation of GQD plus ICIs still requires prospective testing in molecularly defined MSS-CRC patients.

The SCFA axis remains context-dependent. Butyrate can support epithelial integrity, influence macrophage polarization and enhance CD8+ T-cell function in some CRC immunotherapy settings (29, 30). However, it has also been linked to CPT1A-mediated fatty-acid oxidation and reduced anti-PD-1 responsiveness under specific conditions (31). Therefore, the defensible claim is not that more butyrate is always beneficial. Rather, formula-level regulation may coordinate microbial composition, metabolite balance and immune-cell metabolism when this effect is experimentally demonstrated.

Key validation approaches should directly test causality. These include antibiotic-mediated microbiota depletion, fecal microbiota transplantation, targeted metabolite rescue or blockade and CD8+ T-cell dependency testing. Multi-time-point sampling of feces, serum, tumor tissue and immune-cell states would further strengthen mechanistic interpretation. Without such evidence, shifts in Akkermansia, Firmicutes, butyrate-producing bacteria or SCFA abundance should be interpreted as mechanistic signals rather than proof of functional reversal of the microbiota-metabolite barrier.

### 3.2 Antigen-presentation barrier: restoring DC function and T-cell priming

The antigen-presentation barrier is another central reason why MSS-CRC responds poorly to checkpoint blockade. Low neoantigen exposure, MHC-I downregulation, impaired antigen processing and dysfunctional dendritic cells limit productive cytotoxic T-cell priming. In this setting, TCM interventions are most relevant when they improve antigen capture, antigen processing, dendritic-cell maturation, MHC-related presentation or subsequent T-cell activation.

Shenqi Yichang Formula (SQYC) is a representative intervention for this barrier. Reported findings suggest that SQYC improves anti-PD-1 efficacy in MSS-CRC models by regulating dendritic-cell mitophagy through the PINK1-Parkin pathway and enhancing antigen presentation (18). This mechanism is important because mitochondrial quality control in dendritic cells may determine their metabolic fitness, antigen-presenting capacity and ability to initiate T-cell responses.

Several questions still require direct testing. Does tumor control depend on dendritic cells? Are antigen-presentation markers functionally increased? Is CD8+ T-cell priming required for the combined effect? The causal chain should be tested at several levels. These include DC-specific perturbation of PINK1-Parkin signaling, ex vivo antigen-presentation assays and DC-T-cell co-culture. T-cell receptor clonality analysis and spatial profiling would further clarify whether improved antigen presentation leads to productive T-cell responses.

Patients with low DC activation, weak MHC-I/antigen-processing signatures, poor CD8+ T-cell priming or an immune-ignorant phenotype may be suitable for antigen-presentation-oriented formulas. In tumors dominated by stromal exclusion or suppressive myeloid accumulation, however, this strategy is unlikely to be sufficient alone.

### 3.3 Myeloid/Treg-dominant suppressive niche: loosening cellular and metabolic immune suppression

MSS-CRC often contains a suppressive immune network composed of MDSCs, Tregs, M2-like TAMs and inhibitory cytokine circuits. These populations inhibit T-cell activation through multiple routes. These include arginine depletion, IL-10, TGF-beta, reactive oxygen species, checkpoint-associated signaling and metabolic competition. TCM-related effects on these cells are relevant to ICI sensitization only when they reduce suppressive dominance and restore effector function in the context of ICI treatment.

Several TCM interventions and TCM-related reviews report changes in Treg abundance, macrophage polarization, MDSC infiltration or cytokine profiles (32–34). Modified Shenling Baizhu San, for example, has been associated with memory-like Tfh differentiation and B-cell responses in colon cancer models (20). Such immune reorganization may contribute to antitumor activity and, in suitable settings, support checkpoint responsiveness.

Direct myeloid/Treg-targeted ICI sensitization remains less mature than the SCFA-GPR109A or SQYC-DC axes. Many studies measure immune-cell proportions without proving that suppressive cells drive resistance or that their reduction is required for therapeutic benefit. Claims regarding MDSC, Treg or TAM remodeling should therefore remain mechanistic. Stronger evidence would require cell depletion, adoptive transfer, single-cell state mapping or effector-rescue assays.

Informative readouts should capture immune-cell abundance, functional state, spatial location and interaction with effector cells. Single-cell RNA sequencing, spatial transcriptomics, multiplex immunofluorescence and functional cytotoxicity assays can determine whether suppressive niches are dismantled rather than merely numerically reduced.

### 3.4 Immune-exclusion and stromal barrier: from immune-cell abundance to spatial accessibility

Immune exclusion is distinct from immune suppression. In many MSS-CRC tumors, immune cells are present but restricted to stromal regions, invasive margins or CAF-rich compartments. CAF/TGF-beta signaling, extracellular-matrix remodeling, abnormal vasculature and chemokine imbalance can prevent cytotoxic T cells from entering tumor nests. Thus, increased immune-cell abundance is insufficient if spatial access is not restored.

Direct evidence that TCM remodels stromal immune exclusion in MSS-CRC remains limited. However, this barrier defines important validation endpoints. Proposed spatially active formulas should be tested in models that preserve tissue architecture, such as organoid-immune co-culture and orthotopic models. Multiplex imaging and spatial transcriptomics can then be used to measure CD8+ T-cell distance to tumor cells, CAF-rich stroma and myeloid clusters. Candidate readouts include CXCL9/CXCL10, CAF/TGF-beta signatures, vascular normalization markers and extracellular-matrix features.

This barrier refines the positioning of TCM. Formulas that primarily regulate microbiota or systemic metabolism may affect immune exclusion indirectly. In contrast, formulas that modify CAF-associated cytokines, vascular permeability, chemokine gradients or matrix remodeling may deserve priority testing in immune-excluded MSS-CRC models. Such distinctions are essential for biomarker-enriched trial design.

### 3.5 Low tumor immunogenicity barrier: ICD, antigen exposure and secondary immune amplification

Low tumor immunogenicity is a fundamental obstacle in MSS-CRC. Compared with MSI-H/dMMR disease, MSS tumors generally have lower neoantigen load and weaker spontaneous immune recognition. Strategies that increase antigen exposure or induce immunogenic cell death (ICD) may therefore create substrates for checkpoint blockade. ICD is characterized by DAMP release or exposure, including HMGB1, ATP and calreticulin. These signals can promote dendritic-cell recruitment, antigen cross-presentation and secondary T-cell priming (35–37).

Some TCM-derived interventions and natural products induce pyroptosis-like, apoptosis-associated or ICD-related phenotypes in CRC models. These findings are relevant but not sufficient for an ICI-sensitization claim unless they are linked to improved ICI response, increased antigen presentation, CD8+ T-cell dependence and functional immune memory.

The IDO1-kynurenine-AHR axis intersects with this barrier by suppressing effector T-cell function and promoting Treg expansion even when tumor antigens are released (38). TCM interventions that influence tryptophan metabolism, glycolysis, CD36-related fatty-acid metabolism or oxidative stress may therefore affect whether antigen exposure becomes productive immune activation (39). At present, this pathway should be framed as a high-priority hypothesis rather than a mature translational claim.

The framework also clarifies timing. Microbial or metabolic dysregulation may favor induction or priming strategies. Impaired antigen presentation may require DC-oriented formulas. Myeloid/Treg-rich or immune-excluded tumors require validation of suppressive niches and spatial accessibility. Partially activated settings may benefit from maintenance strategies that preserve barrier integrity, microbiota stability and treatment tolerance.

These five barriers are analytically distinct but biologically interconnected. Microbiota-metabolite remodeling can reduce suppressive tone, while antigen-presentation restoration can initiate T-cell priming. Loosening myeloid/Treg niches may permit effector expansion, and reduced immune exclusion may improve tumor access. Increased immunogenicity can provide antigenic material for secondary immune amplification. Together, these links support a stratified approach to TCM-based ICI sensitization.

**Figure 2:**
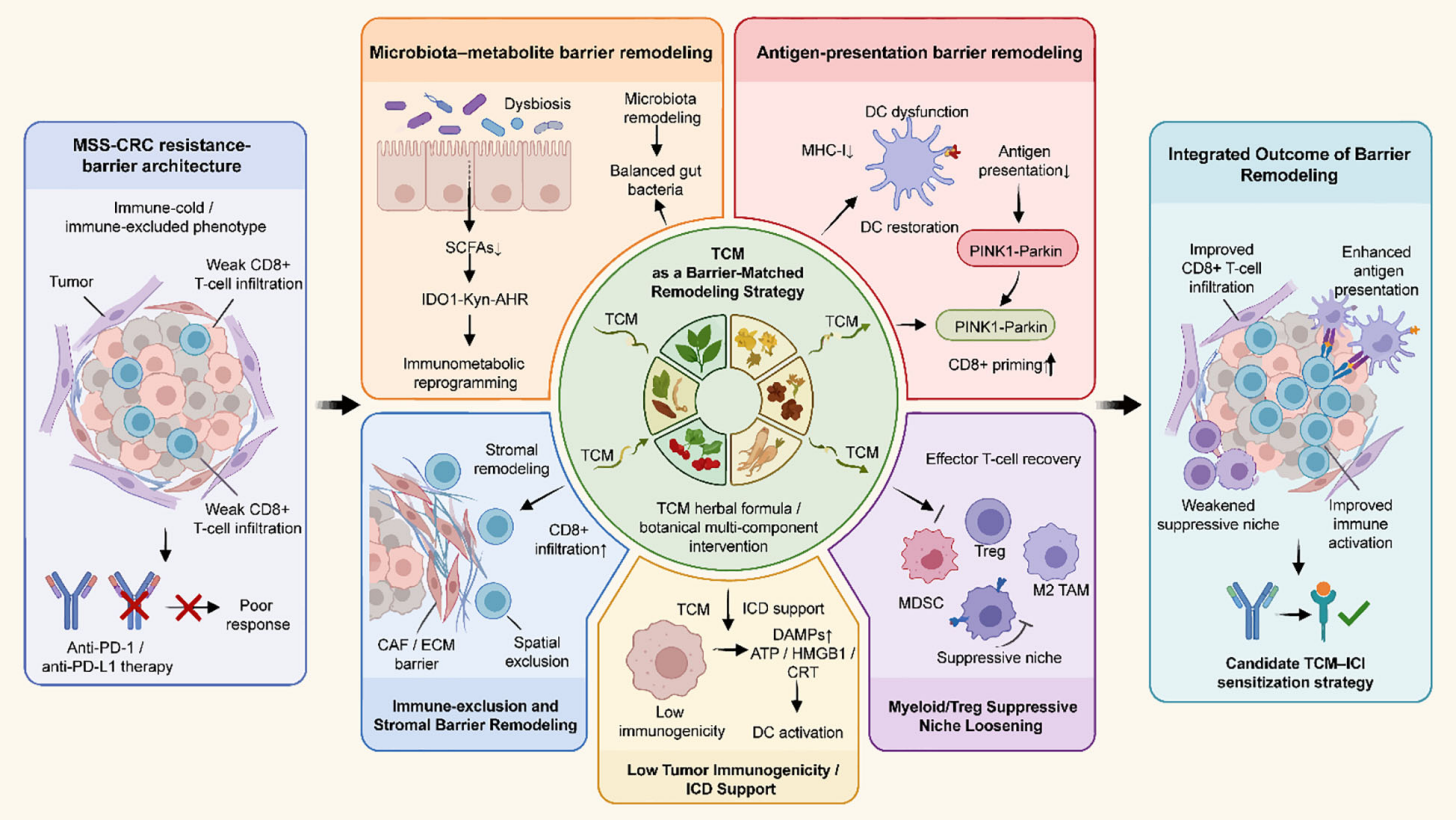
Barrier-based mechanisms of TCM-mediated resistance-barrier remodeling in MSS-CRC. TCM may function as a barrier-matched candidate strategy in MSS-CRC by modulating microbiota–metabolite dysregulation, impaired antigen presentation, suppressive myeloid/Treg niches, immune-exclusion/stromal barriers, and low tumor immunogenicity. These effects may promote immune-permissive remodeling and improve ICI responsiveness, but require formula-specific efficacy evidence, causal validation, quality control, and biomarker-linked readouts. DC, dendritic cell; ICD, immunogenic cell death; MSS-CRC, microsatellite-stable colorectal cancer; TCM, traditional Chinese medicine; Treg, regulatory T cell.

The next stage of research should move from pathway enumeration to barrier-matched validation. Each major resistance barrier should be linked to a corresponding TCM module and to the experimental validation required for a credible sensitization claim (**Figure 2**).

## 4 Representative herbal interventions, evidence hierarchy, and stratification of potentially responsive populations

**Figure 3:**
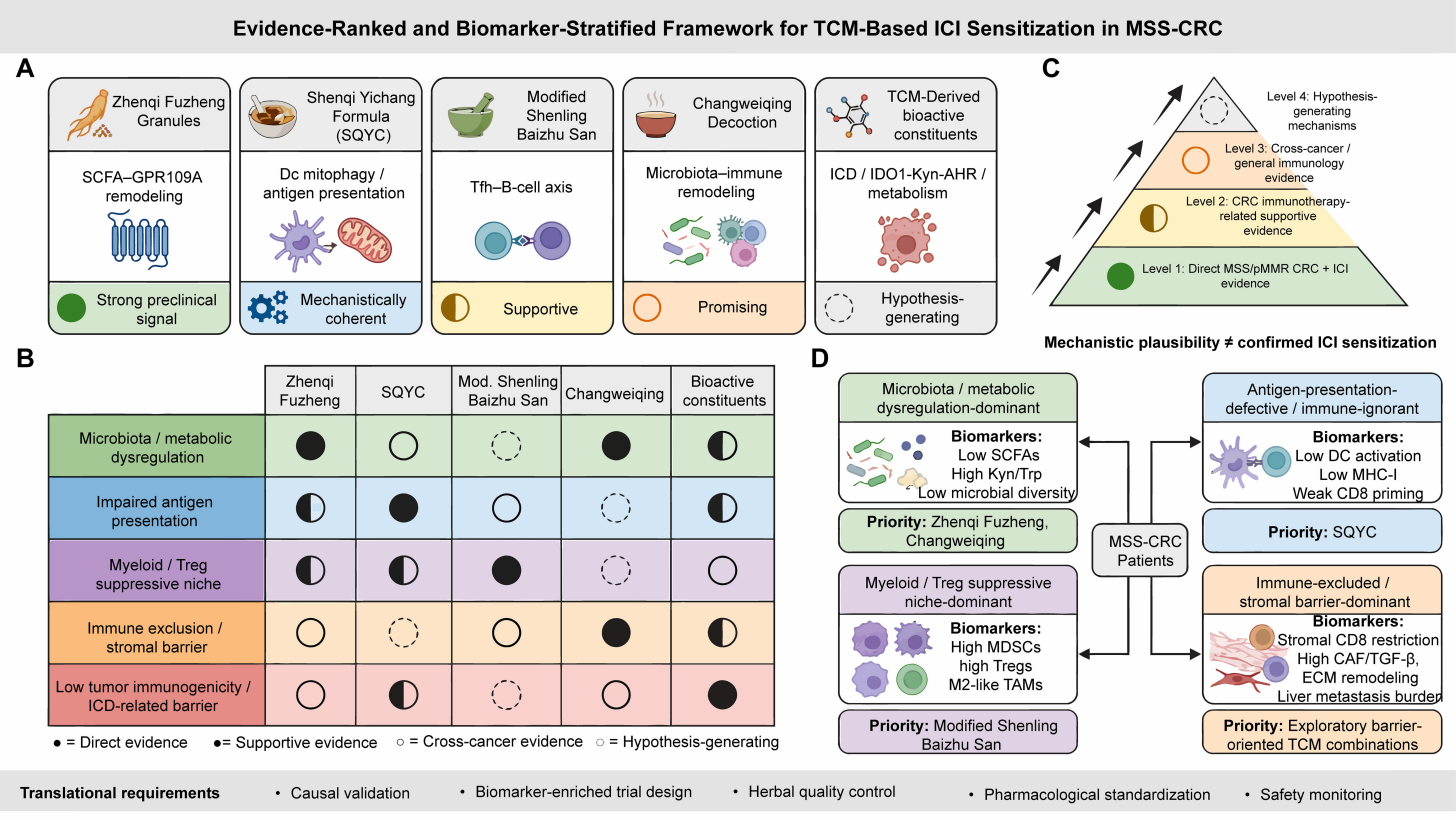
Evidence-ranked and biomarker-stratified framework for TCM-based ICI sensitization in MSS-CRC. **(A)** representative herbal intervention modules and putative barrier-matched mechanisms; **(B)** the matrix-based evidence map; **(C)** the hierarchy of evidence strength; and **(D)** biomarker-defined responsive-population hypotheses and priority intervention routes. CAF, cancer-associated fibroblast; CRC, colorectal cancer; DC, dendritic cell; ICI, immune checkpoint inhibitor; ICD, immunogenic cell death; Kyn/Trp, kynurenine-to-tryptophan ratio; MDSC, myeloid-derived suppressor cell; MSS-CRC, microsatellite-stable colorectal cancer; pMMR, proficient mismatch repair; SCFA, short-chain fatty acid; TAM, tumor-associated macrophage; TCM, traditional Chinese medicine; Tfh, T follicular helper cell; Treg, regulatory T cell.

After defining the mechanistic framework, the next question is which TCM interventions have coherent evidence chains, clearer translational priority and plausible responsive populations. This section first summarizes representative formulas, active constituents and evidence levels. It then discusses functional clustering, multi-barrier mechanisms, comparison with Western sensitization strategies, biomarker- and syndrome-informed stratification, and available clinical-translational evidence for future study design (**Figure 3**).

### 4.1 Representative formulas and active constituents: mechanisms of action and levels of evidence

Representative TCM interventions with relatively coherent evidence in MSS-CRC or CRC immunotherapy-related models are summarized according to formula composition or key constituent information, evidence model and ICI partner, reported dose or treatment duration, immune- and microbiota-related readouts, evidence strength, validation gaps and translational relevance (**Table 1**).

**Table 1:** Structured comparison of representative TCM interventions relevant to barrier-oriented ICI sensitization in MSS-CRC.

Formula/intervention and key information	Evidence model and ICI partner	Reported dose/duration	Main immune/microbiota readouts	Evidence strength	Key validation gaps/priority
GQD: Puerariae Lobatae Radix, Scutellariae Radix, Coptidis Rhizoma and Glycyrrhizae Radix et Rhizoma; key signals include puerarin, baicalin/baicalein, berberine and glycyrrhizic acid (22, 40–42).	CT26 MSS-type CRC model plus anti-PD-1 (22); separate CRC clinical evidence without ICI (54).	GQD 300 mg/kg by gavage for 10 days; anti-PD-1 mAb 250 μg i.p. at 3-day intervals where reported.	Gut microbiota remodeling, metabolic pathway changes, cytokine activation, CD8+ T-cell-related response and intestinal-barrier protection. Multi-barrier signal: microbiota-metabolism-TME-CD8+ axis.	Direct preclinical MSS-type CRC + ICI evidence; clinical non-ICI evidence is supportive.	Needs MSS-CRC clinical testing, microbiota-transfer validation, component exposure assessment and standardized fingerprints.
Zhenqi Fuzheng Granules: Astragali Radix and Ligustri Lucidi Fructus-based preparation; candidate markers include Astragalus polysaccharides/saponins and Ligustrum-related constituents.	CRC immunotherapy-related mouse model plus PD-1 antibody (21).	Exact granule dose, antibody dose, route and duration: NR.	Butyrate-producing bacteria, SCFAs, GPR109A axis, AKT/mTOR/HIF-1α signaling, Warburg-like metabolism and PD-1 response. Multi-barrier signal: microbiota-SCFA-immunometabolism axis.	Direct preclinical formula + ICI evidence.	Requires metabolite rescue/blockade, CD8+ dependency testing, clinical validation, LC-MS/HPLC batch control and syndrome-linked stratification.
SQYC: multi-herb reinforcing formula; candidate marker domains include ginsenosides and Astragalus-related constituents.	MSS-CRC experimental model plus anti-PD-1 antibody (18).	SQYC extract 11.4 g/kg/day; anti-PD-1–5 mg/kg where reported.	PINK1-Parkin-mediated DC mitophagy, antigen-presentation markers and T-cell priming-related endpoints. Potential downstream barrier: T-cell priming and effector expansion.	Direct preclinical MSS-CRC + ICI evidence.	Requires DC-specific perturbation, antigen-presentation assays, CD8+ dependency testing, dose-response analysis and biomarker validation.
Changweiqing/Chang Wei Qing Decoction: intestinal microenvironment-regulating formula; composition and quality markers require standardized reporting.	CRC mouse model plus PD-1 inhibitor (19); CAC model plus PD-1 inhibitor as supportive evidence (56).	Extract dose, PD-1 inhibitor dose and treatment duration: NR.	Gut microbiota diversity, beneficial microbial taxa, immune microenvironment remodeling, intestinal barrier signals and suppressive-niche markers.	Direct preclinical CRC + ICI-related evidence; MSS-specific confirmation remains limited.	Needs MSS/pMMR-focused validation, FMT/metabolite causality, effector-immune linkage, dose clarification and QC reporting.
Modified Shenling Baizhu San/Decoction: spleen-strengthening formula family; modified prescriptions require composition-level reporting.	Colon cancer model without direct ICI combination (20).	No ICI partner in the key cited study; dose/duration should be verified before protocol-level comparison.	Memory-like Tfh differentiation, B-cell responses and immune-supportive TME remodeling.	Supportive CRC evidence; not direct ICI-sensitization evidence.	Needs ICI-combination testing, lymphoid-aggregate functional validation, CD8+ dependency testing and controlled batch profiling.
Active constituents/natural products: berberine, baicalin/baicalein, puerarin, glycyrrhizic acid, ginsenosides and Astragalus-related polysaccharides/saponins (40–48).	Mostly component-level, cross-cancer, pathway-focused or computational evidence.	Compound-specific; PK exposure, tissue distribution and microbiota biotransformation are often underreported.	PD-L1-related signaling, CSN5/STAT3/SPOP pathways, oxidative stress, ICD-like phenotypes, IDO1-Kyn-AHR and metabolic nodes.	Supportive or hypothesis-generating evidence.	Requires compound-specific ICI-combination validation, exposure-response testing, purity reporting, toxicity and interaction assessment.

NR, not reported; DC, dendritic cell; FMT, fecal microbiota transplantation; QC, quality control. Direct ICI-sensitization evidence requires improved ICI efficacy in an MSS/pMMR CRC-relevant setting with barrier-relevant remodeling. Citation numbering follows the current manuscript reference list; Gegen Qinlian Decoction (GQD); Shenqi Yichang Formula (SQYC).

Beyond this summary, interpretation of formula-level evidence also requires attention to the material basis of each intervention. Formula-level efficacy does not directly identify which constituents contribute to immune remodeling or ICI-related effects. GQD provides an illustrative example. It contains Puerariae Lobatae Radix, Scutellariae Radix, Coptidis Rhizoma and Glycyrrhizae Radix et Rhizoma Praeparata cum Melle (40). Representative constituents include puerarin, baicalin/baicalein, berberine-type alkaloids and glycyrrhizic acid. Several of these constituents have immune-relevant activities. Berberine has been reported to reduce tumor-cell PD-L1 expression by inhibiting CSN5-mediated deubiquitination (41). Baicalein and baicalin can suppress IFN-gamma-induced PD-L1 expression through STAT3-related mechanisms and enhance T-cell-mediated tumor-cell killing in experimental systems (42). These observations do not replace formula-level evidence. However, they help explain how individual components may contribute to checkpoint-related immune remodeling.

For other representative formulas, component-level evidence remains less direct. Astragalus-related polysaccharides/saponins, ginsenosides and Ligustrum-related constituents are therefore discussed mainly as candidate quality-marker or mechanism-deconvolution domains rather than as confirmed ICI-sensitizing constituents. Network pharmacology, systems pharmacology, pharmacotranscriptomics and gene-essentiality resources may help prioritize candidate constituents, pathways and target dependencies (43–48). In addition, pharmacokinetic exposure, tissue distribution, metabolism and microbiota-mediated biotransformation of representative constituents should be considered when interpreting formula-specific activity. These computational predictions should be regarded as starting points for experimental validation rather than direct mechanistic proof.

### 4.2 Evidence hierarchy: separating direct sensitization evidence from supportive mechanisms

A critical requirement is to avoid treating all mechanistic observations as equivalent evidence for ICI sensitization. The strongest evidence refers to studies in which a defined formula, active component or integrative modality improves ICI efficacy in an MSS/pMMR CRC-relevant setting. Such studies should also show barrier-relevant immune, metabolic, microbiota or spatial remodeling. This level of evidence is distinct from general antitumor activity or pathway modulation.

By contrast, supportive CRC evidence, cross-cancer evidence, active-ingredient studies and computational analyses mainly provide biological plausibility. These studies can help identify candidate mechanisms, such as microbiota-metabolite regulation, antigen presentation, suppressive immune-cell remodeling or immune-exclusion-related pathways. However, they should not be interpreted as direct proof of MSS-CRC ICI sensitization unless they are experimentally linked to improved ICI response.

An evidence hierarchy is therefore used to separate direct MSS/pMMR CRC plus ICI evidence from supportive CRC evidence, cross-cancer evidence, clinical-translational evidence and hypothesis-generating mechanisms (**Table 2**). This distinction helps clarify what can be concluded from the current literature and what still requires causal and clinical validation.

**Table 2:** Barrier-based evidence hierarchy and validation needs for TCM-based ICI sensitization in MSS-CRC.

Barrier or claim	Direct MSS/pMMR CRC + ICI evidence	Supportive evidence	Key validation needed	Interpretation
Microbiota-SCFA/metabolic remodeling	Zhenqi Fuzheng Granules: SCFA remodeling and improved PD-1 response (21); GQD: enhanced anti-PD-1 activity in MSS-type CRC (22).	SCFA biology and microbiota-metabolite links in CRC/immunotherapy (28–31).	Antibiotic/FMT tests; metabolite rescue or blockade; CD8+ dependency.	Relatively strong preclinical signal; clinical evidence remains exploratory.
DC mitophagy/antigen presentation	SQYC: improved anti-PD-1 response via PINK1-Parkin-associated DC mitophagy (18).	DC dysfunction and impaired antigen presentation are recognized MSS-CRC barriers (11, 35–37).	DC-specific perturbation; antigen-presentation and DC-T-cell assays.	Mechanistically coherent; priority for validation.
Tfh-B-cell axis/lymphoid aggregates	Modified Shenling Baizhu San: Tfh-B-cell responses in colon cancer models (20); direct ICI evidence is limited.	TLS, Tfh and B-cell interactions may support antitumor immunity.	ICI-combination testing; Tfh/B-cell perturbation; spatial mapping.	Promising but immature for ICI-sensitization claims.
Myeloid/Treg/TAM suppressive niche	Formula-specific direct MSS/pMMR CRC plus ICI evidence remains limited.	TCM/CRC studies suggest effects on Tregs, TAMs, MDSCs and suppressive cytokines (32–34).	Cell depletion; single-cell/spatial profiling; effector-rescue assays.	Plausible; should be interpreted cautiously.
ICD, IDO1-Kyn-AHR, CAF/stromal exclusion	Direct TCM plus ICI evidence in MSS-CRC remains sparse.	CRC, cross-cancer and pharmacological studies support these barriers as targets (37–39, 50–52).	Barrier-specific models; pathway blockade; spatial topology assays.	Hypothesis-generating; not yet established.

Direct evidence requires a defined TCM intervention or active component, an MSS/pMMR CRC-relevant model or patient cohort, an ICI-containing design, benefit beyond ICI monotherapy, and barrier-relevant remodeling. Supportive evidence indicates biological plausibility but does not by itself establish ICI sensitization..

### 4.3 Reinforcing, harmonizing, and eliminating-pathogen strategies: functional clustering and combinatorial logic

The available interventions may be broadly grouped into three functional paths. Reinforcing-type formulas tend to restore immune-cell function, improve the metabolic foundation and support host tolerance. Harmonizing-type interventions often act at metabolism-immunity interfaces. Eliminating-pathogen-type formulas may disrupt local barriers, induce ICD or increase tumor immunogenicity. This classification is a functional abstraction rather than a rigid taxonomy.

Within this functional grouping, several representative interventions can be interpreted as potential multi-barrier modulators rather than as single-pathway agents. The strength of evidence varies across interventions. The clearest current examples are GQD and Zhenqi Fuzheng Granules in CRC immunotherapy-related models. GQD may link gut-microbiota remodeling with lipid-metabolic regulation in MSS-type CRC models (22, 28). These changes may further relate to TME reshaping and CD8+ T-cell-related antitumor immunity. Zhenqi Fuzheng Granules may connect butyrate-producing bacteria, SCFA-GPR109A signaling, AKT/mTOR/HIF-1alpha-related metabolic remodeling and improved PD-1 antibody response (21). SQYC is more antigen-presentation-centered, but improved dendritic-cell mitophagy may secondarily affect T-cell priming and effector expansion (18). Electroacupuncture is not a herbal formula, but available MSS-CRC evidence suggests that it may enhance anti-PD-1 activity through TME remodeling and STING-dependent immune activation (49).

These examples indicate that combined mechanisms may emerge when microbiota-metabolic, antigen-presentation and immune-effector barriers are biologically coupled. However, such effects should be interpreted as hypothesis-driven mechanistic signals unless supported by causal perturbation, controlled ICI-combination testing and biomarker-linked validation.

Mechanistically complementary interventions should be selected according to the dominant resistance barrier and TME features of a given subset. Accordingly, this classification should be understood as a hypothesis-driven framework for stratified validation rather than a staged treatment algorithm (**Table 3**).

**Table 3:** Stratified synergistic validation framework for combining TCM with ICIs according to the dominant resistance barrier.

Dominant resistance barrier	Core phenotype	Synergistic direction more suitable for testing	Herbal formulas/settings that may be prioritized	Current evidence status
Microbiota/metabolic dysregulation-dominant	Reduced SCFAs, disturbed Kyn/Trp ratio, amplified peripheral inflammation	Microbiota regulation + metabolic correction	Zhenqi Fuzheng Granules, Changweiqing, etc.	Preclinical signals are available, but multimodel and causal validation are still needed
Immune exclusion/impaired antigen presentation-dominant	Poor CD8+ infiltration, low DC function, prominent CAF/TGF-beta-related stromal barrier	Restoration of antigen presentation + loosening of spatial barriers	Shenqi Yichang Formula and mechanism-matched combination settings	Direct evidence remains limited; priority validation in organoid and spatial-omics settings is warranted
Myeloid suppression/Treg-enriched dominant	Enrichment of MDSCs, Tregs, or M2-like TAMs; poor response to PD-1 monotherapy	Loosening of suppressive networks + amplification of effector T-cell function	Modified Shenling Baizhu San and suppressive-network-oriented combinations	Evidence is currently based mainly on mechanistic plausibility and supportive findings
Maintenance/consolidation exploratory setting	Need to delay re-resistance and preserve tolerance and intestinal barrier homeostasis after initial disease control	Microecological maintenance + systemic support	Maintenance/consolidation cohorts or real-world settings	At present, more suitable as an exploratory direction

This framework is intended to generate research hypotheses and validation pathways and should not be used as a clinical treatment recommendation.

### 4.4 Comparison with current Western sensitization strategies: strengths, limitations, and evidence gaps

Compared with Western sensitization strategies, TCM may offer a system-oriented route for modulating metabolism, immunity and microbiota. These Western strategies include anti-angiogenic or multikinase combinations, chemotherapy, radiotherapy and targeted therapy. However, the clinical benchmark is demanding. IMblaze370 showed that mechanistically rational MEK/PD-L1 blockade does not necessarily improve survival in unselected metastatic CRC (13). REGONIVO suggested activity for regorafenib plus nivolumab (14, 15). However, subsequent pMMR/MSS cohorts showed more modest and population-dependent effects (14, 15). The RIN regimen provided another signal, especially in patients without liver metastases, but it remains early-phase and not generalizable to all MSS-CRC populations (16).

These examples highlight heterogeneous efficacy, site dependence, toxicity and biomarker uncertainty in non-TCM sensitization strategies. They provide the basis for comparing these strategies with TCM-based barrier-oriented combinations (**Table 4**).

**Table 4:** Comparison between TCM-based and commonly used Western sensitization strategies in MSS-CRC immunotherapy.

Strategy type	Representative regimen	Main mechanism	Strengths	Limitations	Evidence level
MEK/PD-L1 combination	Cobimetinib + atezolizumab	Increase T-cell infiltration and inflammatory signaling	Mechanistically rational, phase III tested	No OS benefit over regorafenib in IMblaze370; limits of pathway-based all-comer combinations	Negative phase III (13)
Multikinase/anti-angiogenic + PD-1 or dual ICI	Regorafenib + nivolumab; regorafenib + ipilimumab + nivolumab	Vascular/kinase modulation, myeloid remodeling and T-cell reinvigoration	Clinical activity in selected subgroups	Heterogeneous activity; liver-metastasis dependence; toxicity and biomarker uncertainty	Phase Ib/II; subgroup-dependent (14–16)
Chemotherapy combination	FOLFOX/FOLFIRI-based chemotherapy + ICI	Immunogenic stress, antigen release and partial MDSC reduction	Mature oncology platform	Myelosuppression; potential immune suppression; limited MSS-CRC-specific benefit without stratification	Phase II/III or exploratory
Radiotherapy combination	SBRT or local radiotherapy + PD-1/PD-L1 blockade	DAMP release, antigen exposure and IFN-related signaling	Strong local immune-priming rationale	Narrow applicability; radiation-related injury; abscopal effect remains inconsistent	Preclinical/early clinical
TCM-based combination	Barrier-matched formulas + ICIs	Multi-target modulation of microbiota, metabolism, antigen presentation and suppressive niches	System-level regulation and potential tolerability advantage	Lower evidence level; complex composition; causal validation and QC required	Preclinical; hypothesis-driven translation

Accordingly, TCM is best positioned as a barrier-matched synergistic modulator, not as a substitute for established therapies. A candidate intervention becomes translationally meaningful only when it can answer three questions. Which barrier does it target? Which patient subset is likely to benefit? How can its effect be causally validated? Multi-target regulation and potential tolerability advantages should remain hypotheses until they are supported by quality control, pharmacological standardization and biomarker-linked outcomes.

### 4.5 Biomarker- and syndrome-informed stratification of potentially responsive populations

The practical value of a barrier-oriented framework depends on its ability to generate testable stratification hypotheses. Because MSS-CRC is biologically heterogeneous, TCM-ICI strategies are unlikely to be equally informative in unselected populations. Modern biomarkers should first be used to define the dominant immune-resistance barriers.

Several biomarker-defined contexts may be informative. First, patients with pronounced microbial dysbiosis, reduced SCFAs or elevated Kyn/Trp ratios may be more suitable for microbiota- and metabolism-centered strategies (11, 19, 21, 24, 25, 38, 50). Second, patients with immune-excluded tumors and prominent CAF/TGF-beta-associated stromal barriers may require approaches that improve spatial T-cell access (10, 11, 51, 52). Third, patients with marked myeloid suppression, Treg/MDSC enrichment and poor responsiveness to PD-1 monotherapy may provide settings for testing TCM-mediated remodeling of suppressive immune niches (9, 32, 33, 52).

These stratification concepts remain exploratory and are not sufficient for direct clinical decision-making. Nevertheless, baseline subgrouping may reduce signal dilution in unselected cohorts. A practical marker set may include peripheral microbiota and metabolic indicators, local tumor immune indicators, genomic and disease-anatomical variables, and integrated clinical factors. Particular attention should be paid to liver metastasis and treatment-line heterogeneity. Clinical experience with multikinase/ICI combinations suggests that MSS-CRC immunotherapy responsiveness may be strongly influenced by metastatic distribution (14–16).

TCM syndrome differentiation may add a complementary host-state layer to this biomarker-based framework. Spleen deficiency or qi deficiency may be explored as host-state indicators of reduced systemic reserve, nutritional vulnerability and impaired treatment tolerance. Dampness-heat accumulation may be associated with chronic intestinal inflammation, dysbiosis and metabolic disturbance. Phlegm-dampness/stasis or blood-stasis/toxin patterns may suggest metabolic stress, stromal remodeling or vascular abnormality. They may also be explored in relation to spatial immune restriction. These associations should be interpreted as exploratory links between TCM clinical phenotypes and modern immune-resistance biomarkers, rather than as fixed biological equivalences.

Future MSS-CRC immunotherapy studies may record TCM syndrome patterns using predefined criteria and analyze them together with molecular, immune, microbiota, metabolic and spatial readouts. Such integration may help generate patient-stratification and formula-matching hypotheses, in which candidate formulas are tested according to both dominant immune-resistance barriers and standardized TCM syndrome patterns, while preserving modern biomarkers as the primary basis for trial design.

### 4.6 Available clinical and translational evidence for TCM-ICI combinations in MSS-CRC

Clinical and translational evidence should therefore be interpreted in layers rather than treated as equivalent proof of TCM-ICI efficacy. These layers include clinical evidence of TCM-related or integrative modalities within ICI-containing regimens, clinical CRC evidence of herbal formula-mediated microbiota or immune modulation without ICIs, and direct preclinical formula plus anti-PD-1 evidence in MSS-type CRC models. These evidence layers are summarized according to study design/model, disease status, intervention, main findings and interpretation (**Table 5****).**

**Table 5:** Available clinical and translational evidence related to TCM or TCM-related interventions combined with ICI-based strategies in MSS-CRC.

Evidence category	Study design/model	Disease status	Intervention	Main finding	Interpretation
Direct clinical integrative-modality evidence	Phase II, single-arm clinical study (53)	Refractory MSS-mCRC after >=2 prior regimens	Electroacupuncture + fruquintinib + sintilimab	Feasible regimen with acceptable safety and antitumor activity; ORR, DCR, PFS and OS signals reported	Clinical feasibility signal; EA-specific contribution requires controlled validation because a VEGFR TKI was included
Clinical herbal-formula evidence without ICI	Clinical CRC study (54)	CRC patients; not an ICI-combination trial	Gegen Qinlian Decoction	Immune modulation and intestinal-barrier protection through gut microbiota-related mechanisms	Supports clinical biological plausibility for microbiota-barrier regulation, but does not prove ICI sensitization
Direct preclinical formula + ICI evidence	MSS-type CRC mouse model (22)	CT26 MSS-type CRC model	GQD + anti-PD-1	Enhanced PD-1 blockade with gut microbiota and TME remodeling	Strong translational rationale for microbiota-barrier testing; clinical confirmation is needed
Direct preclinical formula + ICI evidence	MSS-CRC model (18)	MSS-CRC experimental model	SQYC + PD-1 monoclonal antibody	Improved anti-PD-1 efficacy through PINK1-Parkin-mediated DC mitophagy and antigen presentation	Supports antigen-presentation-oriented validation and DC/CD8+ dependency testing
Direct preclinical formula + ICI evidence	CRC immunotherapy-related model (21)	CRC model with PD-1 antibody treatment	Zhenqi Fuzheng Granules + PD-1 antibody	Enhanced PD-1 efficacy via SCFA-GPR109A immunometabolic remodeling	Supports microbiota-metabolite-defined stratification; patient-level validation is required
Direct preclinical TCM-related modality + ICI evidence	MSS-CRC mouse models (49)	Multiple MSS-CRC models	Electroacupuncture + anti-PD-1	Enhanced antitumor immunity through TME remodeling and STING-dependent mechanisms	Provides mechanistic rationale for EA + ICI strategies, but does not substitute for herbal-formula evidence

Direct clinical evidence for herbal formula plus ICI therapy in molecularly confirmed MSS-CRC remains scarce. Clinical, non-ICI clinical and preclinical evidence in this table should be interpreted as different levels of translational support rather than equivalent clinical validation.

An early phase II single-arm study in refractory MSS-mCRC reported the feasibility, acceptable safety and antitumor activity of electroacupuncture combined with fruquintinib and sintilimab (53). This study provides a clinical signal for a TCM-related modality within an ICI-containing regimen. However, because the regimen also included a VEGFR TKI, the independent contribution of electroacupuncture requires controlled validation.

For herbal formulas, the current evidence is mainly translational rather than definitive clinical proof. GQD has shown microbiota- and intestinal-barrier-related effects in CRC patients without ICI treatment (54). Preclinical data also suggest that GQD can enhance PD-1 blockade in MSS-type CRC models (22). These evidence streams are complementary but should not be conflated. Future studies should connect formula exposure, syndrome patterns, microbiota-metabolic biomarkers, immune-barrier remodeling and ICI-based clinical outcomes within the same patient population.

## 5 Strengthening the preclinical evidence base for TCM-based ICI sensitization in MSS-CRC

Current evidence supports the possibility that TCM may improve MSS-CRC responsiveness to ICIs in selected contexts, but the evidence base remains uneven. Direct sensitization evidence is concentrated in a limited number of formulas or integrative modalities. Broader claims regarding microbiota regulation, immunometabolic remodeling, antigen presentation, myeloid/Treg suppression or immune reprogramming rely largely on indirect CRC, cross-cancer or mechanism-oriented evidence. The priority is to define which formula, active component or modality modulates which barrier, in which syndrome- or biomarker-defined host state, and with what degree of causal support.

### 5.1 Why antitumor enhancement is not equivalent to true ICI sensitization

A recurrent limitation is the interpretation of improved tumor inhibition after a combined intervention as proof of ICI sensitization. A formula may suppress tumor growth through cytotoxic, anti-inflammatory, anti-angiogenic, metabolic or microbiota-mediated effects without restoring checkpoint-dependent immunity. A stronger sensitization claim requires an ICI arm and combination superiority over both monotherapies in an MSS/pMMR-relevant model. It should also show recovery of barrier-relevant immune functions and include causal testing through cell depletion, pathway perturbation, microbiota transfer or metabolite rescue/blockade. Otherwise, tumor size, immune-cell proportions, microbial abundance and pathway markers should be treated as mechanistic signals rather than definitive evidence.

### 5.2 What current formula-specific studies can legitimately support

Several representative interventions are now linked to interpretable biological processes. These include Zhenqi Fuzheng Granules with SCFA-related immunometabolic remodeling, Shenqi Yichang Formula with dendritic-cell mitophagy and antigen presentation, GQD with microbiota/TME remodeling and CD8+ T-cell-related antitumor immunity, modified Shenling Baizhu San with the Tfh-B-cell axis, Changweiqing with microbiota and microenvironmental regulation, and electroacupuncture with STING-dependent remodeling of the tumor immune microenvironment (18–22). These studies begin to connect TCM-related interventions with barrier-relevant mechanisms in MSS-CRC or related immunotherapy settings. They also illustrate why multi-barrier signals should be interpreted within formula-specific evidence chains rather than generalized across all TCM interventions.

Their limitations are equally important. Most studies still show pathway, microbial, metabolite or immune-cell changes without fully establishing the sequence from intervention exposure to barrier remodeling and improved checkpoint responsiveness. Clinical studies are even more limited, especially for herbal formulas combined with ICIs in molecularly confirmed MSS-CRC. The literature is therefore sufficient to identify mechanistic entry points, but not yet sufficient to define robust formula-specific therapeutic claims.

### 5.3 Matching validation systems to dominant resistance barriers

Validation systems should match the claimed mechanism. Microbiota-metabolite mechanisms require depletion, transplantation or targeted metabolite interrogation; dendritic-cell or antigen-presentation mechanisms require direct functional assays; and immune-exclusion or stromal mechanisms require models that preserve tissue architecture and spatial context.

Because MSS-CRC is biologically heterogeneous, future work should move beyond conventional cell-line assays and simplified subcutaneous systems toward organoid-immune co-culture, orthotopic or humanized models, and spatially informative analyses.

### 5.4 From mechanistic plausibility to candidate translational signals

Mechanistic studies should generate candidate translational signals, not pathway lists. Microbiota-metabolic claims should include SCFAs, Kyn/Trp indices, microbial diversity or metabolite-associated immune changes. Antigen-presentation claims should include DC activation and functional priming. Suppressive-niche claims should include Treg, MDSC, macrophage or spatial immune-distribution parameters.

**Figure 4:**
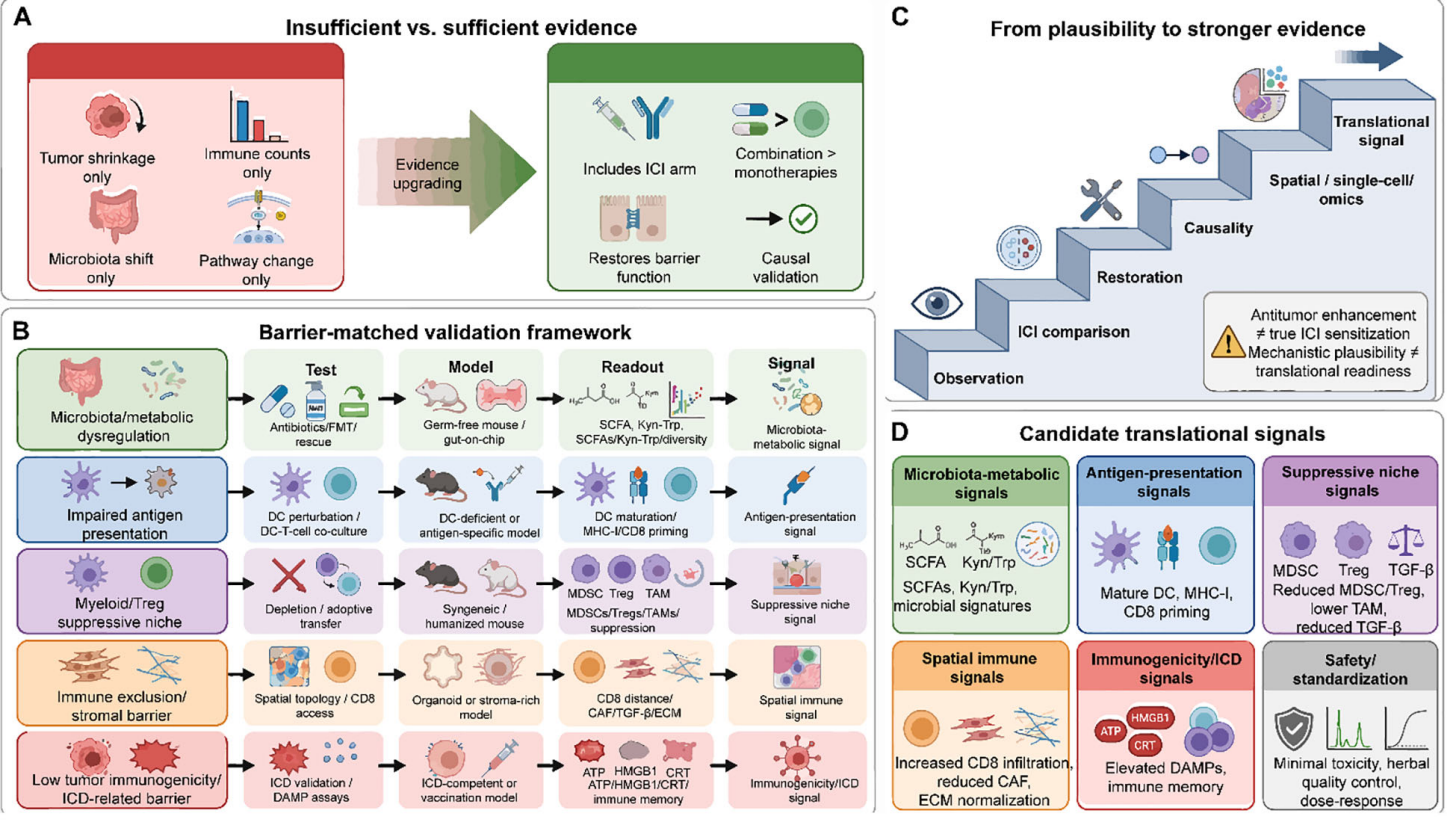
Strengthening the preclinical evidence base for TCM-based ICI sensitization in MSS-CRC. **(A)** Insufficient evidence is distinguished from the minimum criteria required to support a true ICI-sensitization claim. **(B)** Barrier-matched framework linking major resistance barriers to appropriate causal tests, model systems, functional readouts, and candidate translational signals. **(C)** Stepwise progression from mechanistic plausibility to stronger preclinical evidence, highlighting that antitumor enhancement is not equivalent to true ICI sensitization. **(D)** Representative translational signals generated by strengthened preclinical studies, including microbiota–metabolic, antigen-presentation, suppressive niche, spatial immune, immunogenicity/ICD, and safety/standardization-related readouts.

Single-cell and spatial approaches are particularly valuable because increased immune-cell abundance does not necessarily indicate effective sensitization. Stronger preclinical evidence will come from defining which cell states, suppressive compartments and spatial immune distributions are altered by a given formula. A strengthened preclinical evidence framework should therefore distinguish antitumor enhancement from true ICI sensitization, match causal tests to dominant barriers, and generate candidate translational signals (**Figure 4**).

## 6 Clinical translation of TCM-based ICI sensitization in MSS-CRC: evaluation frameworks, trial design, and key bottlenecks

For TCM to become a credible candidate strategy in MSS-CRC immunotherapy, preclinical signals must be translated into clinically interpretable evidence. The key issue is whether formula-specific effects can be measured in patients, linked to meaningful endpoints, and reproduced across biologically heterogeneous settings.

### 6.1 Integrated evaluation beyond single-endpoint efficacy

Evaluation of TCM combined with ICIs should integrate clinical outcomes, mechanistic responses and host-state assessment. Clinical outcomes include ORR, PFS, OS and treatment-related toxicity (55). Mechanistic responses should include immune-cell composition, cytokine/chemokine profiles, microbiota parameters and metabolic indicators, such as SCFAs and Kyn/Trp ratio. Host-state measures may include syndrome characteristics, functional status and nutritional condition.

This integrated framework reduces interpretive ambiguity. Clinical benefit without biological remodeling risks overattribution to immune sensitization. Mechanistic change without clinical improvement may reflect biological activity without therapeutic relevance. Therefore, the contribution of TCM should be judged by convergence between clinical outcomes and barrier-relevant biological changes.

### 6.2 Biomarker-informed stratification and barrier-oriented trial design

Baseline characterization should cover molecular and anatomical factors. These include MSI/MMR status, RAS/BRAF mutation status, tumor sidedness, prior treatment exposure, liver metastatic status and extrahepatic metastatic burden. Baseline assessment should also include microbiota-metabolic features, spatial immune phenotype, performance status, nutritional status and relevant TCM syndrome characteristics. TCM syndrome characteristics should be recorded using predefined criteria. They should be treated as exploratory host-state variables rather than substitutes for molecular or immune biomarkers.

Endpoint selection should follow the same logic. ORR, PFS, OS and irAEs should be complemented by prespecified mechanistic readouts. These may include CD8+ T-cell infiltration and spatial distribution, Treg/MDSC abundance, macrophage polarization, microbial diversity, SCFAs, Kyn/Trp metabolism, ctDNA dynamics and barrier-specific tissue biomarkers.

Three trial formats appear particularly relevant. Window-of-opportunity studies can define early biological effects before extensive treatment-induced confounding. Biomarker-enriched randomized studies can test whether a formula is active in the barrier-defined subgroup for which it is mechanistically intended. Maintenance or consolidation studies can evaluate whether TCM preserves tolerance, microecologic stability or response durability after initial disease control. This design may be especially relevant in patients with controlled extrahepatic disease or limited liver involvement.

### 6.3 Major translational bottlenecks

A major bottleneck is the gap between preclinical plausibility and clinical evidence. Although immunometabolic and microbiota-oriented mechanisms are increasingly discussed, MSS-CRC-specific clinical data remain sparse. Direct clinical evidence for herbal formulas combined with ICIs is still lacking. Existing clinical signals from integrative modalities or non-ICI herbal studies should therefore be used to generate hypotheses rather than to establish efficacy.

Additional bottlenecks include mechanistic deconvolution of complex formulas and methodological heterogeneity across the literature. Multi-component formulas may be therapeutically advantageous. However, they complicate active-component attribution, pathway prioritization, batch consistency and cross-study comparability. Variability in formula composition, quality control, treatment schedule, sampling strategy, endpoints and negative-reporting practices further limits reproducibility.

### 6.4 Herbal quality control and pharmacological standardization

Pharmacological standardization is a prerequisite for evaluating TCM-ICI combinations. In these studies, immune or microbiota remodeling can be interpreted meaningfully only when the tested formula is chemically traceable and reproducible.

Future studies should first report the basic material information of the tested formula. This includes botanical source, voucher or supplier information, processing method and extraction procedure. They should also provide exposure- and batch-related information, including extract yield, dosage conversion, administration route, batch number, fingerprint chromatogram and quantitative markers for representative constituents. Chemical equivalence between decoctions, granules, extracts and purified fractions should not be assumed without analytical confirmation. For multi-herb formulas, the proportion of each herb, quality markers for major botanical drugs, storage stability and batch-to-batch consistency should also be documented.

Quality-control data should be linked to barrier-specific pharmacological readouts. LC-MS/MS, HPLC/UPLC fingerprinting, metabolomics and pharmacotranscriptomic signatures can help connect formula exposure with immune, microbiota and metabolic endpoints. Relevant endpoints include SCFAs, Kyn/Trp ratio, DC activation, CD8+ T-cell infiltration, Treg/MDSC abundance, CAF/TGF-beta signaling and spatial immune distribution. Such integration is important because changes in these readouts may otherwise be difficult to distinguish from batch variation, background treatment effects or nonspecific biological activity.

Clinical translation further requires safety-oriented standardization. Liver and renal function, gastrointestinal tolerance, irAEs and potential interactions with chemotherapy, targeted therapy or ICIs should be monitored together with formula quality-control information. Standardization will not eliminate the complexity of TCM formulas. However, it can improve the interpretability, reproducibility and cross-study comparability of TCM-ICI combination studies. Therefore, QC information should be prespecified and reported alongside efficacy, mechanistic and safety endpoints in future TCM-ICI studies.

### 6.5 Safety, interpretability, and the next translational step

Safety evaluation should be embedded in trial design from the outset. Potential risks include amplification of irAEs, disturbance of gastrointestinal tolerance or microbiota homeostasis, and pharmacokinetic interactions with chemotherapy or targeted agents. Safety monitoring, interaction assessment and standardized reporting should therefore be incorporated early in clinical development.

Several evidence limitations should be acknowledged. First, direct MSS-CRC plus ICI plus herbal-formula studies remain scarce. As a result, part of the mechanistic discussion relies on supportive CRC, cross-cancer or microbiota/immunometabolism evidence. Second, non-TCM sensitization trials show that biologically plausible combinations can fail or benefit only selected subgroups (13, 15, 16). Third, early integrative-modality evidence, such as electroacupuncture combined with fruquintinib and sintilimab, remains hypothesis-generating and requires controlled validation in larger prospective cohorts (53).

There are also methodological limitations. The evidence classification used here improves interpretive transparency, but it is not a formal quantitative grading system. It cannot substitute for systematic review, meta-analysis or guideline-level assessment. TCM translation also remains uncertain without formula-specific quality control, pharmacological standardization, biomarker-enriched enrollment and causal validation across microbiota, metabolite, immune, spatial and syndrome-related endpoints.

In this context, ‘sensitization’ denotes a candidate or hypothesis-generating concept rather than an established clinical effect. It is not treated as established unless improved ICI efficacy is directly demonstrated in an MSS/pMMR CRC-relevant model or clinical cohort. Mechanistic changes in tumor size, microbiota composition, cytokines or immune-cell proportions alone were not interpreted as definitive ICI sensitization.

**Figure 5:**
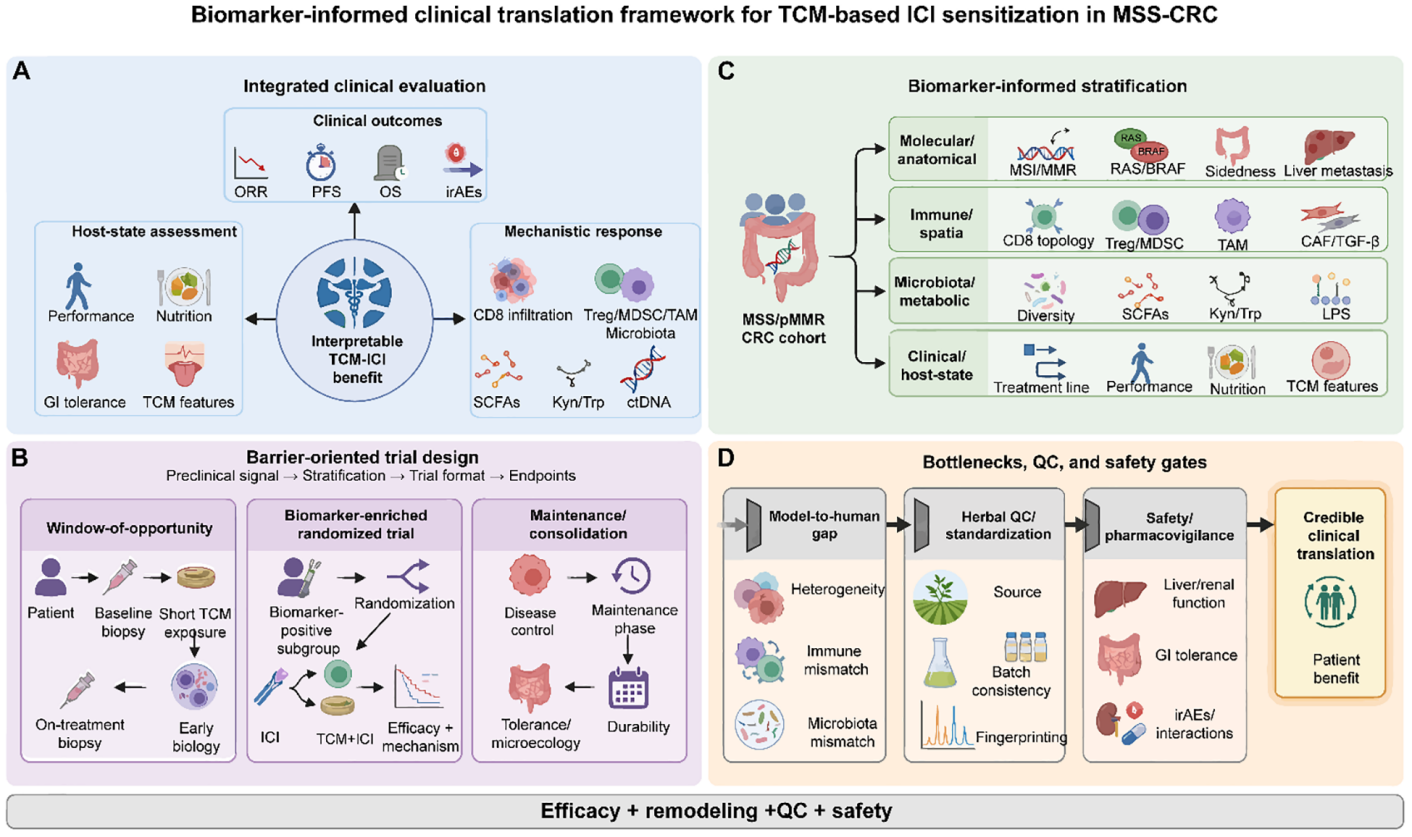
Biomarker-informed clinical translation framework for TCM-based ICI sensitization in MSS-CRC. **(A)** Integrated clinical evaluation combining clinical outcomes, mechanistic responses, and host-state assessment to define interpretable TCM–ICI benefit. **(B)** Barrier-oriented trial design, including window-of-opportunity studies, biomarker-enriched randomized trials, and maintenance/consolidation strategies. **(C)** Biomarker-informed stratification of MSS/pMMR CRC patients across molecular/anatomical, immune/spatial, microbiota/metabolic, and clinical/host-state dimensions. **(D)** Key translational gates involving model-to-human gaps, herbal quality control and standardization, and safety/pharmacovigilance before credible clinical translation.

Progress will depend less on expanding candidate formulas than on improving interpretability. Future studies should clarify which formulas or components should be tested, in which biological and TCM syndrome contexts, with which biomarkers, and under which quality-control and safety standards. These considerations can be integrated into a biomarker-informed clinical translation framework that connects evaluation endpoints, patient stratification, trial design, quality control and safety monitoring (**Figure 5**).

## 7 Conclusions and perspectives

MSS-CRC resistance to immune checkpoint blockade is sustained by a multilayered barrier architecture. Key barriers include low tumor immunogenicity, impaired antigen presentation, suppressive myeloid and regulatory immune niches, immune exclusion, and microbiota-metabolic dysregulation. Within this framework, TCM should not be viewed as a broadly applicable ICI-enhancing intervention. Instead, it should be positioned as a candidate barrier-oriented and syndrome-informed modulatory strategy for selected biological and host-state contexts.

The most plausible directions include microbiota-metabolite remodeling, partial restoration of antigen presentation, and attenuation of myeloid/Treg-dominant suppressive niches. Other relevant directions include loosening of stromal immune-exclusion structures, enhancement of tumor immunogenicity, and support of host immune-metabolic tolerance. Preclinical signals from GQD and Zhenqi Fuzheng Granules illustrate the possibility of multi-barrier modulation. However, these combined mechanisms still require causal and clinical validation.

The current evidence remains uneven. Relatively direct MSS-CRC or CRC immunotherapy-related evidence is concentrated in a limited number of formulas or integrative modalities. Many claims remain supported mainly by indirect evidence. These include claims related to IDO1-Kyn-AHR signaling, CAF-associated immune exclusion, myeloid remodeling, ICD-related mechanisms and active components. Much of this evidence comes from supportive CRC studies, cross-cancer observations or mechanistic plausibility. Therefore, TCM-based ICI sensitization should not yet be regarded as an established therapeutic strategy.

A defensible claim requires formula-specific evidence showing benefit beyond ICI monotherapy. It should also include barrier-relevant immune or metabolic remodeling, causal validation, component-level characterization and reproducible biomarker-linked readouts. Future studies should move from broad descriptions of multi-target regulation toward disciplined, barrier-matched validation.

Microbiota-metabolic claims should be tested using antibiotic depletion, fecal microbiota transplantation and metabolite rescue or blockade. Antigen-presentation claims should be supported by dendritic-cell functional assays and CD8+ T-cell priming readouts. Immune-exclusion or suppressive-niche claims require models that preserve spatial context. These may include organoid-immune co-culture, orthotopic or humanized models, single-cell analysis, spatial transcriptomics and multiplex imaging. In parallel, clinical translation should avoid uncontrolled all-comer enrollment and adopt biomarker-informed designs. These designs should integrate molecular and anatomical features, microbiota-metabolic indicators, spatial immune phenotypes, host-state variables and standardized TCM syndrome characteristics.

Herbal quality control, pharmacological standardization and safety monitoring must also be integrated into the evidence chain. Future studies should systematically report formula composition, botanical source, processing, extraction and batch consistency. They should also include fingerprint chromatograms, quantitative markers, dose conversion, drug-interaction assessment and safety monitoring. Safety evaluation should cover liver and renal function, gastrointestinal tolerance and immune-related adverse events. Without these standards, even biologically plausible TCM-ICI combinations will remain difficult to interpret, reproduce or translate.

In summary, TCM-based ICI sensitization in MSS-CRC remains promising but early. Its future value will depend less on expanding the number of candidate formulas than on answering four questions. Which formulas, active components or integrative modalities act on which resistance barriers? Which molecularly and syndrome-stratified patient subsets are most relevant? Which measurable biomarkers should be used? Which quality-control and safety conditions are required? If supported by rigorous causal validation and biomarker-enriched clinical studies, TCM may evolve from an empirical adjunct into a precisely positioned, barrier-matched translational module for selected MSS-CRC populations.

## Glossary

CRC: Colorectal cancer
MSS-CRC: Microsatellite-stable colorectal cancer
MSI-H: Microsatellite instability-high
dMMR: Deficient mismatch repair
pMMR: Proficient mismatch repair
ICI: Immune checkpoint inhibitor
PD-1: Programmed cell death protein 1
PD-L1: Programmed death-ligand 1
CTLA-4: Cytotoxic T-lymphocyte-associated protein 4
TME: Tumor microenvironment
TCM: Traditional Chinese medicine
SCFA: Short-chain fatty acid
Kyn/Trp: Kynurenine-to-tryptophan ratio
IDO1: Indoleamine 2,3-dioxygenase 1
AHR: Aryl hydrocarbon receptor
DC: Dendritic cell
Treg: Regulatory T cell
TAM: Tumor-associated macrophage
MDSC: Myeloid-derived suppressor cell
CAF: Cancer-associated fibroblast
Tfh: T follicular helper cell
ICD: Immunogenic cell death
DAMP: Damage-associated molecular pattern
FMT: Fecal microbiota transplantation
scRNA-seq: Single-cell RNA sequencing
QC: Quality control
irAE: Immune-related adverse event
ctDNA: Circulating tumor DNA
ORR: Objective response rate
PFS: Progression-free survival
OS: Overall survival
GQD: Gegen Qinlian Decoction
EA: Electroacupuncture
TKI: Tyrosine kinase inhibitor
STING: Stimulator of interferon genes.
